# Construction of non-polar mutants in *Haemophilus influenzae *using FLP recombinase technology

**DOI:** 10.1186/1471-2199-9-101

**Published:** 2008-11-11

**Authors:** Erin Tracy, Fang Ye, Beth D Baker, Robert S Munson

**Affiliations:** 1 Center for Microbial Pathogenesis in The Research Institute at Nationwide Children's Hospital, Columbus, OH, USA; 2 The Center for Microbial Interface Biology and Department of Pediatrics, The Ohio State University, Columbus, OH, USA

## Abstract

**Background:**

Nontypeable *Haemophilus influenzae *(NTHi) is a gram-negative bacterium that causes otitis media in children as well as other infections of the upper and lower respiratory tract in children and adults. We are employing genetic strategies to identify and characterize virulence determinants in NTHi. NTHi is naturally competent for transformation and thus construction of most mutants by common methodologies is relatively straightforward. However, new methodology was required in order to construct unmarked non-polar mutations in poorly expressed genes whose products are required for transformation. We have adapted the lambda red/FLP-recombinase-mediated strategy used in *E. coli *for use in NTHi.

**Results:**

A cassette containing a spectinomycin resistance gene and an *rpsL *gene flanked by FRT sites was constructed. A PCR amplicon containing 50 base pairs of DNA homologous to the 5' and 3' ends of the gene to be disrupted and the cassette was generated, then recombineered into the target NTHi gene, cloned on a plasmid, using the lambda recombination proteins expressed in *E. coli *DY380. Thus, the gene of interest was replaced by the cassette. The construct was then transformed into a streptomycin resistant NTHi strain and mutants were selected on spectinomycin-containing growth media. A plasmid derived from pLS88 with a temperature sensitive replicon expressing the FLP recombinase gene under the control of the *tet *operator/repressor was constructed. This plasmid was electroporated into the NTHi mutant at the permissive temperature and FLP expression was induced using anhydrotetracycline. The recombinase recognizes the FRT sites and eliminates the antibiotic cassette by site-specific recombination, creating the unmarked non-polar mutation. The plasmid is cured by growth of cells at the restrictive temperature.

**Conclusion:**

The products of the genes in the NTHi *pilABCD *operon are required for type IV pilus biogenesis and have a role in transformation. We demonstrated the utility of our methodology by the construction of a non-polar *pilA *mutation in NTHi strain 2019 and complementation of the mutation with a plasmid containing the *pilA *gene. Utilization of this approach allowed us to readily generate unmarked non-polar mutations in NTHi genes.

## Background

Nontypeable *Haemophilus influenzae *(NTHi) is a gram-negative bacterium, which is a major cause of otitis media [1,2]. The organism also causes pneumonia and other respiratory tract diseases in humans [1,2]. Type IV pili (Tfp) mediate adherence, twitching motility, and play a role in transformation (reviewed by Craig et al [3]). We previously demonstrated that NTHi produce Tfp under defined environmental conditions. These Tfp are responsible for twitching motility, Tfp-mediated adherence and contribute to biofilm development [4-6]. The products of the *pilABCD *gene cluster play a role in Tfp biogenesis [4,7,8]. The *pilA *gene is predicted to encode the major pilin subunit. The PilB protein is homologous to hexameric secretion ATPases and the PilC protein has homology to inner membrane proteins required for Tfp pilus assembly in other organisms. PilD is predicted to be the prepilin peptidase. These genes are in an operon, which necessitates the construction of non-polar mutants in order to carefully define the role of each gene product.

In NTHi, non-polar mutants have been constructed using the non-polar kanamycin resistance cassette designed by Menard et al [9], see for example Mason et al [10]. This kanamycin resistance gene is promoter-less; thus, transcription is driven from the promoter of the operon. The level of transcription must therefore be at a level sufficient to confer a kanamycin-resistant phenotype to the mutant under normal growth conditions. Alternatively, a non-polar mutant can be constructed in two steps. First, a mutant is constructed using standard methodologies [11] in which a gene has been interrupted with a cassette containing both a selectable and counter-selectable marker. Then, DNA containing an in-frame deletion of the gene of interest together with flanking chromosomal DNA 5' and 3' of the deleted gene is transformed into the mutant. Mutants containing the in-frame deletion are isolated by selection against the counter-selectable marker. Neither of these methodologies is suitable for use with genes whose products are required for transformation since the genes are poorly expressed under normal growth conditions and mutants deficient in the expression of these genes cannot be transformed. We thus adapted the methodologies employed by Wanner and coworkers [12,13] to construct non-polar mutations in NTHi.

## Results and discussion

### Construction of a non-polar mutant in the NTHi *pilA *gene

Mutations have been engineered into the *E. coli *chromosome using the lambda phage recombinase to catalyze the site-specific insertion of a PCR amplicon containing 50 bp homology arms and a cassette flanked by FRT (FLP recombinase target) sites, replacing the gene of interest [12,13]. Given the restriction barriers present in NTHi strains, it was necessary to perform the insertional inactivation of the NTHi genes of interest by cloning the gene together with flanking sequences onto a plasmid, then insertionally inactivating the gene in *E. coli *before moving the mutation into NTHi using the natural transformation system [11]. *E. coli *strain DY380 has previously been used for engineering plasmids using the products of the phage lambda recombinase genes [14]. This strain is lysogenized with a defective lambda phage that expresses the recombinase genes under the control of the temperature sensitive λ*cI*857 repressor and thus is a suitable host for recombineering [15]. In order to construct a *pilA *mutation in NTHi, the *pil *gene cluster was first amplified from NTHi strain 2019, then cloned into pGEM-T Easy and transformed into *E. coli *strain DY380 to form strain DY380(pRSM2855).

**Figure 1:**
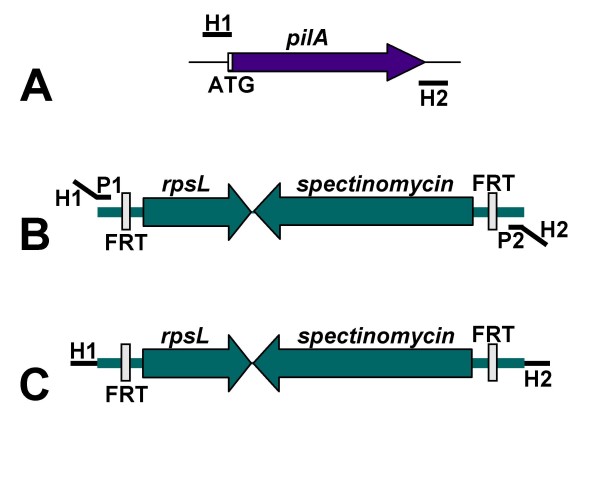
**Generation of a PCR amplicon for insertion/deletion mutagenesis of the *pilA *gene**. In Panel A, the *pilA *gene from NTHi strain 2019 is shown. Homology arm 1 (H1) contains 50 base pairs of DNA 5' to and including the ATG start codon of *pilA*. Homology arm 2 (H2) contains the complement of the last 21 base pairs of the *pilA *gene and 29 base pairs immediately downstream of the *pilA *gene. In Panel B, the template region of pRSM2832 is shown. Primer H1P1 contains the sequence from H1 and 20 base pairs of DNA homologous to the 5' end of the cassette (P1). Primer H2P2 contains the sequence from H2 and 20 base pairs of DNA homologous to the 3' end of the cassette. PCR amplification of pRSM2832 with primers H1P1 and H2P2 (primers 5 and 6) yielded the product shown in Panel C.

**Figure 2:**
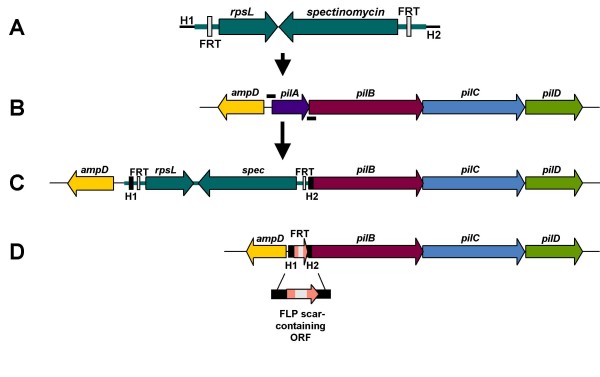
**Construction of a non-polar *pilA *mutant**. The PCR amplicon from Figure 1 is shown in Panel A. The cloned *pilABCD *region of pRSM2855 is shown in Panel B. The amplicon was electroporated into *E. coli *DY380(pRSM2855) and the lambda recombinase genes were induced by temperature shock. Spectinomycin-resistant clones were selected. The insert region of plasmid pRSM2857 is shown in Panel C. Plasmid pRSM2857 was linearized and transformed into NTHi strain 2019 *rpsL*; spectinomycin-resistant clones were isolated. NTHi strain 2019 *rpsL*Δ*pilA*::spec-*rpsL*_Ng _was saved, then transformed with pRSM2947 at the permissive temperature; kanamycin resistant clones were isolated. Expression of the FLP recombinase resulted in the loss of the spectinomycin resistance gene-*rpsL*_Ng _cassette; growth at 37°C resulted in the loss of the plasmid. The *pil *region of NTHi strain 2019 *rpsL*Δ*pilA *is depicted in Panel D.

The plasmid pKD13 contains the kanamycin resistance gene from Tn5 flanked by FRT sites recognized by the FLP recombinase [13]. We modified pKD13 so that it contained a spectinomycin resistance gene; in NTHi, spectinomycin provides a strong selectable marker with little background. The normal allele of an *rpsL *gene from *Neisseria gonorrhoeae *(designated *rpsL*_Ng_) was also added to the construct to provide a counter selectable marker [16]. This plasmid was designated pRSM2832. Employing pRSM2832 as a template, a PCR amplicon was generated using the strategy of Baba et al [12]. The 5' primer was designed to include 50 nt 5' to and including the ATG start codon of the *pilA *gene. The 3' primer was designed to contain the complement of the last 21 nt including the stop codon of the *pilA *gene. Both primers contained regions homologous to the cassette containing the spectinomycin resistance gene and the *rpsL*_Ng _gene (Figure 1). The amplicon (Figure 2A) was electroporated into *E. coli *strain DY380(pRSM2855) after heat shock and spectinomycin-resistant clones were isolated. A plasmid containing the anticipated insertion/deletion mutation in the *pilA *gene was saved as pRSM2857 (Figure 2C).

NTHi strain 2019 is a well-characterized isolate from a patient with chronic bronchitis [17-19]. The pRSM2857 insert was separated from the plasmid backbone by digestion with NcoI and NsiI, then transformed into a streptomycin-resistant derivative of NTHi strain 2019, designated NTHi strain 2019 *rpsL*, using the MIV methodology [11]. A strain containing the anticipated insertion/deletion mutation in the *pilA *gene was saved as NTHi strain 2019 *rpsL*Δ*pilA*::spec-*rpsL*_Ng_. The next step in the construction was the removal of the cassette, which is flanked by FRT sites, by expression of the FLP recombinase.

**Figure 3:**
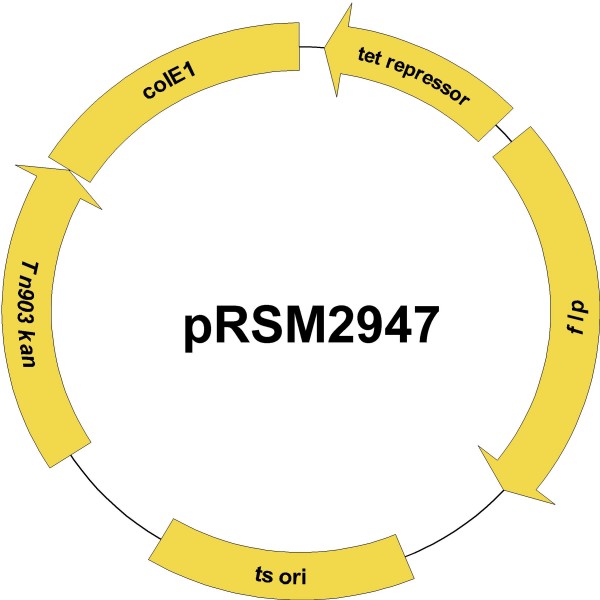
**Map of plasmid pRSM2947**. The plasmid contains: the FLP recombinase gene under the control of the *tet *regulatory system; a temperature sensitive replicon suitable for use in NTHi; a ColE1 origin that is functional in *E. coli *and a kanamycin resistance marker.

We constructed a temperature sensitive derivative of pLS88, a plasmid that replicates in NTHi and cloned the FLP recombinase gene into this construct under control of the *tet *operator/repressor (Figure 3). This plasmid, designated pRSM2947, was electroporated into NTHi strain 2019 *rpsL*Δ*pilA*::spec-*rpsL*_Ng _and kanamycin resistant clones were isolated. One clone was saved as NTHi strain 2019 *rpsL*Δ*pilA*::spec-*rpsL*_Ng _(pRSM2947). Expression of the FLP recombinase was induced in this strain, then clones were isolated at 37°C on streptomycin-containing medium. One streptomycin-resistant clone was saved as NTHi strain 2019 *rpsL*Δ*pilA *for further characterization. The *pil *region in the mutant is represented in Figure 2D.

### The NTHi strain 2019 *rpsL*Δ*pilA *mutant was not transformable; the phenotype was complemented by the *pilA *gene

The product of the genes in the *pil *gene cluster is required for transformation using the MIV methodology [4]. Transformability of the Δ*pilA *mutant was tested using a modification of the MIV methodology [4]. In order to generate a DNA substrate for transformation, we cloned, then insertionally inactivated, the *H. influenzae *strain Rd *cya *gene with a non-polar kanamycin cassette to form pRSM2948 (data not shown). Plasmids were digested with NotI. The linear DNA fragment containing the interrupted *cya *gene was transformed into the NTHi strain 2019 *rpsL*Δ*pilA *and strain 2019 *rpsL *using the modified MIV methodology. Transformants were selected on chocolate agar containing kanamycin. Transformation efficiency (number of transformants/μg of DNA) is shown in Table 1. Approximately 5 × 10^5 ^transformants/μg of DNA was obtained with strain 2019 *rpsL*, while transformants were not detected with the mutant under these conditions. We cloned the *pilA *gene into a shuttle vector forming pRSM2848 and electroporated the construct into NTHi strain 2019 *rpsL*Δ*pilA*. We then tested the transformability of the complemented strain using the interrupted *cya *gene as described above. Approximately 1.5 × 10^5 ^transformants/μg of DNA was obtained with the complemented strain (Table 1).

**Table 1:** Transformation of strain 2019 derivatives

**Number of transformants/μg of DNA**			
**Strains**			

	2019 *rpsL*	2019 *rpsL*Δ*pilA*	2019 *rpsL*Δ*pilA *(pRSM2848)

Experiment #1	5.4 × 10^5^	ND	1.3 × 10^5^
Experiment #2	4.8 × 10^5^	ND	1.7 × 10^5^

**Strains**			

	2019	2019 *pilA*::ΩKm2	2019 *pilA*::ΩKm2 (pRSM2848)

Experiment #3	3.9 × 10^3^	ND	ND
Experiment #4	5.6 × 10^3^	ND	ND

ND is not detected.

Although the putative functions of the *pilB*, *pilC *and *pilD *genes make it likely that the products of these genes are required for transformation, we demonstrated that a polar mutation in the *pilA *gene could not be complemented with plasmid pRSM2848, the plasmid expressing the *pilA *gene. A polar *pilA *mutant, designated 2019 *pilA*::ΩKm2, was constructed by insertion of the ΩKm2 cassette into the *pilA *gene as described [4]. As anticipated, this mutant is not transformable and the mutation could not be complemented with the cloned *pilA *gene (Table 1).

## Conclusion

We have developed a new methodology for the construction of mutants in NTHi. This methodology was developed to overcome technical issues related to the construction of non-polar mutants in poorly expressed genes whose products are required for transformation. However, the method is straightforward and robust. We have successfully constructed over 20 mutants using this method and now construct all *H. influenzae *mutants in our laboratory using this method. The methodology should also be applicable to other naturally transformable members of the *Pasteurellaceae *family that support the replication of pLS88 derivatives.

## Methods

### Bacterial strains, plasmids and growth conditions

NTHi strain 2019 is a well characterized strain from a patient with chronic bronchitis [17-19]. NTHi strain 2019 *rpsL *was constructed by Apicella and coworkers. This strain contains a mutation resulting in a K43R modification in RpsL; the strain is resistant to 1 mg of streptomycin/ml. Both strains were kindly provided by Dr. Michael Apicella. NTHi strains were grown on chocolate agar or in Brain Heart Infusion broth supplemented with 2 μg of NAD/ml and 2 μg of heme/ml (sBHI). Growth media were supplemented with spectinomycin, kanamycin, chloramphenicol or streptomycin at 200 μg/ml, 20 μg/ml, 1 μg/ml or 1 mg/ml, respectively, when appropriate. *E. coli *strains were grown on LB broth or agar. Growth media were supplemented with spectinomycin, kanamycin, chloramphenicol or ampicillin at 50 μg/ml, 20 μg/ml, 30 μg/ml or 50 ug/ml, respectively, when appropriate. Strain DY380 was grown at 32°C. A complete list of strains and plasmids used in this study is provided [see Additional file 1].

### Construction of a template plasmid containing a cassette with a spectinomycin resistance gene and an *rpsL *gene flanked by FRT sites

The plasmid pKD13 has been used as a template plasmid for the construction of a complete set of *E. coli *strains with mutations in each non-essential gene [12]. We modified this vector by replacing the kanamycin resistance gene with a spectinomycin resistance gene and the *rpsL*_Ng _gene. The plasmid pSpecR [20] was digested with EcoRV and the EcoRV fragment containing the spectinomycin resistance gene was cloned into the EcoRV site of pWSK30 [21]. A plasmid with the correct restriction map was saved as pRSM2790. The counter selectable marker, *rpsL*_Ng_, was amplified by PCR using genomic DNA purified from *N. gonorrhoeae *strain FA1090 and primers 1 and 2 containing BamHI and NotI sites at the 5' end, respectively (Table 2). The amplicon was digested with NotI and BamHI, then cloned into the plasmid pRSM2790 that had been digested with these enzymes. A plasmid with the correct insert was identified and saved as pRSM2830. The kanamycin cassette from pKD13 was eliminated by PCR amplification of a portion of pKD13 using primers 3 and 4 which face outward from inside the FRT sites. The spectinomycin resistance gene-*rpsL*_Ng _cassette was isolated from the pRSM2830 by digestion with SacI and XhoI and cloned into the pKD13-derived PCR product. Clones were selected on LB-agar containing spectinomycin. The plasmid from a spectinomycin-resistant clone was characterized by restriction digestion and sequencing, then saved as pRSM2832.

**Table 2:** Oligonucleotide primers

**Primer**	**Sequence**
primer 1	GC**GGATCC**CCGACTGATTGTGAGGGATGTCGG

primer 2	GC**GCGGCCGC**CCGACTGATTGTGAGGGATGTCGG

primer 3	GC**GAGCTC**GCATCGCCTTCTATCGCCTTCTTG

primer 4	GC**CTCGAG**CAGCCCTTGCGCCCTGAGTG

primer 5	CTTTTCACAATGTTGTCGCTAACAAAGGCTTAATAAAAGGAAAATGAATGATTCCGGGGATCCGTCGACC

primer 6	ACGCTGAGTATGAAGTAAAGCATAGCTCGTCATTTTGTGACACTTCTGCATGTAGGCTGGAGCTGCTTCG

primer 7	GCGCGTCGACAACCAATAAGGAAATA

primer 8	CGAGGCAATGGATCAACAGAAG

primer 9	GGGCGTTTATCGAAGTGAGG

primer 10	TCAACCCCTAGCCAAAGAC

primer 11	CGCGGATCCTGCCGCCTGTTTTTCCTGCTCATT

primer 12	CTGTTATCCTTAAATCTCGCTTATTAGGTGTGCTTGTATTTCTTGGG

primer 13	CCCAAGAAATACAAGCACACCTAATAAGCGAGATTTAAGGATAACAG

primer 14	CGC**GGATCC**GCGGCCGCGTGACACGACGATGCTAAA

primer 15	ATATAAT**GCGGCCGC**TACGGTTATCCACAGAATCA

primer 16	ATATAAT**GCGGCCGC**CTCACTGATTAAGCATTGG

primer 17	CGC**GGATCC**CGATGGGTGGTTAACTCGAC

primer 18	CGC**GGATCC**ACAGGACGGGTG

primer 19	GC**ACGCGT**TCGTCAAATATTACGGATATTAA

primer 20	GC**ACGCGT**TCATTGTGTGACACTTCCG

Restriction endonuclease cleavage sites are shown in bold.

### Amplification of the cassette

Since lambda recombinase requires less than 50 bp of homology for efficient recombination, PCR primers of approximately 70 nt in length were designed using the strategy of Baba et al [12]. The 5' PCR primer (primer 5) had a 50 nt homology arm (H1) including the sequence upstream of *pilA *as well as the *pilA *start codon and the 20 nt sequence 5'-ATTCCGGGGATCCGTCGACC-3' (P1) which is complementary to sequence 5' of the spectinomycin resistance gene-*rpsL *cassette (Figure 1). The 3' primer (primer 6) contained the complement of the last 21 nt of the *pilA *gene including the termination codon and 29 nt downstream (H2) as well as the 20 nt sequence 5'-TGTAGGCTGGAGCTGCTTCG-3' (P2) which is complementary to sequence 3' of the spectinomycin resistance gene-*rpsL*_Ng _cassette. A PCR amplicon was generated using these primers together with pRSM2832 as the template. Amplification was performed in a 50 μl reaction containing 1 U Phusion High Fidelity DNA Polymerase (New England Biolabs), 25 ng pRSM2832 DNA, 0.4 μM of each primer, and 200 μM dNTPs under the following conditions: 98°C for 30 seconds, 30 cycles of 98°C for 10 seconds, 51°C for 20 seconds, 72°C for 3 minutes, and a final extension of 72°C for 10 minutes. The amplicon thus contains the spectinomycin resistance gene-*rpsL *cassette flanked by the homology arms H1 and H2.

### Construction of a *pilA *mutant in NTHi strain 2019 *rpsL *containing the spectinomycin resistance gene-*rpsL*_Ng _cassette

The *pilABCD *gene cluster of NTHi strain 2019 as well as 227 bp 5' of the *pilA *gene was amplified by PCR from genomic DNA purified from NTHi strain 2019 using primers 7 and 8 and cloned into pGEM-T Easy (Promega) (Figure 2B). The nucleotide sequence of the NTHi strain 2019 *pilABCD *gene cluster is 94% identical to the gene cluster in strain 86-028NP (Genbank Accession # CP000057). Plasmid DNA was purified, verified by restriction digestion, and sequenced. A clone with the correct sequence was identified and saved as pRSM2855. Plasmid pRSM2855 was transformed into the *E. coli *strain DY380 at 32°C and clones were selected on LB agar containing ampicillin. Strain DY380 contains a defective lambda prophage with a temperature sensitive repressor [14]. In this strain, the lambda recombinase genes are actively transcribed after temperature shock at 42°C.

To construct a plasmid containing a marked deletion of the *pilA *gene, 200 ng of PCR product containing the spectinomycin resistance gene-*rpsL*_Ng _cassette was electroporated into *E. coli *DY380(pRSM2855) that had been heat shocked at 42°C for 15 minutes prior to preparation of electrocompetent cells as described by Lee et al [14]. Clones were then selected at 32°C on LB agar containing spectinomycin. A plasmid containing a deletion of the *pilA *gene with an insertion of the cassette was identified and saved as pRSM2857 (Figure 2C).

Plasmid pRSM2857 was digested with NcoI and NsiI to release the insert from the pGEM-T Easy vector backbone, then transformed into NTHi strain 2019 *rpsL *using the MIV methodology [11]. Clones were selected on chocolate agar supplemented with spectinomycin. Putative mutants were screened for streptomycin sensitivity on chocolate agar containing streptomycin. The genotype of presumptive mutants was further characterized by PCR amplification of the genomic region using primers 9 and 10, followed by sequencing.

### Construction of the FLP Recombinase containing plasmid, pRSM2947

Deletion of the cassette to generate the non-polar mutation in NTHi strain 2019 *rpsL *requires the activity of the FLP recombinase. We constructed a temperature sensitive plasmid containing the FLP recombinase gene under the control of the *tet *promoter/repressor system. Briggs and Tatum previously reported the construction of a plasmid with a temperature sensitive replicon suitable for use in members of the *Pasteurellaceae *family [22]. This plasmid has the same replicon as the plasmid pLS88 [23]. A point mutation of G to A at base 2196 of pLS88 was introduced into a fragment of pLS88 containing the replicon and kanamycin resistance gene using overlap PCR. Briefly, amplicons 1 and 2 corresponding to NT 1715 to 2220 and 2174 to 4093 of pLS88 were generated with primers 11 and 12 or primers 13 and 14, respectively, using pLS88 DNA as template. Amplicon 3 was generated with primers 11 and 14, which both contain BamHI sites, using amplicons 1 and 2 as template. The amplicon was digested with BamHI, and self-ligated. The ligation mixture was transformed into *H. influenzae *strain Rd by electroporation. Plasmid-containing strains were selected by growth at 32°C on chocolate agar containing kanamycin. Strains containing the correct plasmid were identified by screening for growth at 32°C, but absence of growth at 37°C on chocolate agar containing kanamycin. A temperature sensitive plasmid was saved as pRSM2865. This intermediate plasmid is 2.3 Kb in size and contains unique NotI and BamHI sites. The presence of the mutation in pRSM2865 was confirmed by DNA sequencing. Although pLS88 is stably maintained in *E. coli*, pRSM2865 could not be maintained in *E. coli*, even when grown at 32°C. Thus, for convenience, a fragment of pUC19 containing the ColE1 replicon was amplified with primers 15 and 16; both primers contain a NotI site. The resulting amplicon was digested with Not I, then cloned into NotI-digested pRSM2865. After transformation into *E. coli *strain DH5α, a plasmid with the correct restriction map was saved as pRSM2866. Using pFT-A [24] as template and primers 17 and 18, which contain BamHI sites, an amplicon was generated which contained the FLP recombinase gene under control of the *tet *promoter/repressor system flanked by BamHI sites. The amplicon was digested with BamHI and the product ligated to BamHI-digested pRSM2866. The ligation mixture was transformed into DH5α and clones selected on LB agar containing kanamycin. A plasmid with the correct restriction map was identified and saved as pRSM2947 (Figure 3).

### Construction of *pilA *mutants in NTHi strain 2019 background

In order to construct an in-frame, non-polar deletion of the cloned *pilA *gene in NTHi strain 2019 *rpsL*, plasmid pRSM2947 was transformed by electroporation into NTHi strain 2019 *rpsL*Δ*pilA*::spec-*rpsL*_Ng _and transformants were selected at 32°C on chocolate agar containing kanamycin. Clones were grown at 32°C in sBHI broth supplemented with kanamycin until a final absorbance (600 nm) of 0.30 was reached. The FLP recombinase was then induced with 200 ng of anhydrotetracycline/ml for 2 hours, shaking (180 rpm) at 32°C. Unmarked mutants were then selected by growth at 37°C on chocolate agar supplemented with streptomycin. Mutants were then screened for loss of both spectinomycin and kanamycin resistance genes and the genotype was verified by PCR and sequencing (Figure 2D). Although convenient, the streptomycin counterselection is not necessary. After induction of the FLP recombinase using our methodology, the cassette is resolved in approximately 2% of the clones; thus, it is possible to identify mutants by screening. A detailed protocol for mutant construction in NTHi strains is provided [see Additional file 2].

A polar mutation in the *pilA *gene of strain 2019 was constructed as described [4]. The mutant is designated 2019 *pilA*::ΩKm2.

### Complementation of the *pilA *mutation in NTHi strains 2019 *rpsL*Δ*pilA *and 2019 *pilA*::ΩKm2

The plasmid pPIL1 containing the NTHi *pilABCD *gene cluster [4] was PCR amplified with primers 19 and 20, which face outward from the 3' end of *pilA *and the 3'end of *pilD*. The primers both contain MluI sites on their 5' ends. The amplicon was digested with MluI, self-ligated, and transformed into *H. influenzae *strain Rd. The resulting plasmid contained the pSPEC1 backbone, the *pilA *gene, and a small ORF containing the 5' portion of the *pilB *gene cloned in-frame with the 3' end of the *pilD *gene. A clone with the correct restriction map was sequenced, then saved as pRSM2848. The plasmid pRSM2848 was then transformed into NTHi strains 2019 *rpsL*Δ*pilA *and 2019 *pilA*::ΩKm2.

### Cloning and insertional inactivation of the *cya *gene

We cloned and insertionally inactivated the *cya *gene. This interrupted gene was used to test the strains for their ability to be transformed. The *cya *gene from *H*. *influenzae *strain Rd and approximately 1 Kb of flanking DNA 5' and 3' to the gene were amplified and cloned into pGEM-T Easy essentially as described for construction of pRSM2855. A clone with the correct restriction map was saved as pRSM2921. The non-polar kanamycin cassette [9] was cloned into KpnI- and BamHI-digested pRSM2921 as a KpnI to BamHI fragment. After ligation and transformation, a plasmid containing the insertionally inactivated *cya *gene was identified and saved as pRSM2948. The cloned *cya *gene was also insertionally inactivated by cloning a BamHI fragment containing the chloramphenicol cassette from pUCΔEcat into BglII-digested pRSM2921. As plasmid containing the insertionally inactivated *cya *gene was saved as pRSM3004.

### Determination of transformation efficiency

Transformation efficiency was determined in strains 2019 *rpsL*, 2019 *rpsL*Δ*pilA *and 2019 *rpsL*Δ*pilA*(pRSM2848). DNA from NotI-digested pRSM2948 was incubated with cells treated to induce competence using the modified MIV methodology; transformants were identified on chocolate agar plates containing kanamycin. Similarly, transformation efficiency was determined in strains 2019 *pilA*::ΩKm2 and 2019 *pilA*::ΩKm2(pRSM2848) using linearized pRSM3004 as above, except that transformants were selected on chloamphenicol-containing medium.

## Authors' contributions

ET and RSM designed the strategy and drafted the manuscript. FY constructed pRSM2947. BB cloned and insertionally inactivated the NTHi *cya *gene. ET was responsible for generation of the template plasmid, construction of the mutants and the transformation experiments. All authors read and approved the final manuscript.

## Supplementary Material

Additional file 1 **Bacterial strains and plasmids.** This table contains a complete list of strains and plasmids used in this study. Click here for file

Additional file 2 **Protocol for construction of mutants in NTHi.** A detailed protocol is presented to aid investigators in the use of this technology. Click here for file
