# Supplementary material for: Construction of non-polar mutants in Haemophilus influenzae using FLP recombinase technology
Source: BMC Mol Biol. 2008 Nov 11;9:101. doi: 10.1186/1471-2199-9-101 (PMC2625361; doi:10.1186/1471-2199-9-101)
Supplement: Additional file 1 — Bacterial strains and plasmids. This table contains a complete list of strains and plasmids used in this study. [file 1471-2199-9-101-S1.pdf]

Bacterial strains and plasmids

Strain or Plasmid	Relevant Genotype	Source
E. coli		
DH5α	strain used for general cloning procedures	Invitrogen
DY380	DH10B derivative containing a defective λ prophage; *red*, *bet* and *gam* genes are controlled by the temperature-sensitive λ*cI*857 repressor	[14]
EC100D *pir*$^{+}$	host for propagation of plasmids containing the R6Kγ origin of replication	Epicentre
H. influenzae		
2019	Nontypeable *H. influenzae* strain from a patient with chronic bronchitis	Michael Apicella
2019 *rpsL*	Streptomycin resistant derivative of NTHi 2019	Michael Apicella
2019 *rpsL* Δ*pilA*::spec-$rpsL_{Ng}$	Derivative of NTHi 2019 *rpsL* with an insertion/deletion mutation in the *pilA* gene	this study
2019 *rpsL*Δ*pilA*	Derivative of NTHi 2019 *rpsL* with a non-polar mutation in the *pilA* gene	this study
2019 *pilA*::ΩKm2	Derivative of NTHi 2019 with a polar mutation in the *pilA* gene	this study
**Plasmids**		
pUC19	cloning vector, source of the ColE1 origin	New England Biolabs
pGEM-T Easy	cloning vector	Promega
pWSK30	low copy number cloning vector	[21]
pLS88	*Haemophilus ducreyi* plasmid; useful as a shuttle vector	[23]
pSpecR	source of the spectinomycin resistance gene	[20]
pUC18K	source of the non-polar kanamycin resistance cassette	[9]
pUCΔEcat	source of the chloramphenicol	[25]
pJRS102.0	source of the ΩKm2 cassette	[26]
pKD13	template plasmid used for mutant construction in *E. coli*; contains R6Kγ origin of replication	[13]

pFT-A	source of the FLP recombinase gene under control of the *tet* regulatory system	[24]
pPIL1	shuttle vector containing the *pilABCD* gene cluster from NTHi strain 86-028NP	[4]
pRSM2790	spectinomycin resistance gene from pSpecR cloned into pWSK30	this study
pRSM2830	normal allele of the *Neisseria gonorrhoeae rpsL* gene in pRSM2790	this study
pRSM2832	pKD13 derivative used as a template plasmid for mutant construction in NTHi	this study
pRSM2848	derivative of pPIL1 lacking the *pilB*, *pilC* and *pilD* genes	this study
pRSM2855	*pil* gene cluster from NTHi 2019 cloned into pGEM-T Easy	this study
pRSM2857	*pilA*::spec insertion/deletion mutation of pRSM2855	this study
pRSM2865	plasmid containing the origin of replication and kanamycin-resistance gene from pLS88. The origin of replication contains a point mutation conferring a temperature-sensitive phenotype	this study
pRSM2866	derivative of pRSM2865 containing the ColE1 origin from pUC19	this study
pRSM2921	*cya* gene and flanking DNA cloned into pGEM-T Easy	this study
pRSM2947	derivative of pRSM2866 containing the FLP recombinase gene from pFT-A (Figure 3)	this study
pRSM2948	derivative of pRSM2921 containing an insertion/deletion mutation of *cya*	this study
pRSM3004	derivative of pRSM2921 containing the chloramphenicol cassette in the *cya* gene	this study
