# Supplementary material for: Construction of non-polar mutants in Haemophilus influenzae using FLP recombinase technology
Source: BMC Mol Biol. 2008 Nov 11;9:101. doi: 10.1186/1471-2199-9-101 (PMC2625361; doi:10.1186/1471-2199-9-101)
Supplement: Additional file 2 — Protocol for construction of mutants in NTHi. A detailed protocol is presented to aid investigators in the use of this technology. [file 1471-2199-9-101-S2.pdf]

Construction of unmarked non-polar NTHi mutants

Primer Design

The 5' primer is a 70-mer designed to contain a 50 nt homology arm (H1) including the sequence upstream of the gene to be deleted as well as the start codon and the 20 nt sequence 5'-ATTCCGGGGATCCGTCGACC-3' (P1) which is complementary to sequence 5' of the spectinomycin resistance gene-$rpsL_{\mathrm{Ng}}$ cassette (Figure 1).

The 3' primer is a 70-mer designed to contain the complement of the last 21 nt of the gene to be deleted including the termination codon and 29 nt downstream (H2) as well as the 20 nt sequence 5'-TGTAGGCTGGAGCTGCTTCG-3' (P2) which is complementary to sequence 3' of the spectinomycin resistance gene-$rpsL_{\mathrm{Ng}}$ cassette (Figure 1).

Amplification of the spec-*rpsL* cassette with 50bp homology arms

pRSM2832 (25ηg)
5' primer (20μM)
3' primer (20μM)
dNTPs (10mM)
5X Phusion Buffer HF
1U of Phusion polymerase (New England Biolabs)
dH_20 to 50μl

PCR Conditions

1) 98ºC – 30 seconds
2) 98ºC – 10 seconds
3) 51ºC – 20 seconds
4) 72ºC – 2 minutes
5) Repeat steps 2-4, 30 cycles
6) 72ºC – 10 minutes
7) 4ºC – hold

Deletion/insertion of the gene of interest in the plasmid clone

1. Transform plasmid clone containing the gene of interest and approximately 1Kb of flanking DNA 5' and 3' of the gene into *E.coli* strain DY380 at 32ºC.

2. Inoculate 5ml of L-Broth, supplemented with the appropriate antibiotic, with a DY380 colony from the plate. Incubate at 32ºC, 225rpm, overnight.

3. Dilute overnight culture into 50ml of L-Broth containing the appropriate antibiotic and grow to an OD at 600ηm of ~0.05-0.1.

4. Incubate at 32ºC, 225rpm, until the OD at 600ηm reaches approximately 0.6.

5. Transfer culture to a shaking (225rpm) 42ºC H_20 bath, for 15 minutes.

6. Place cells on ice for approximately 5 minutes. Transfer cells into a 14ml round bottom tube.

7. Harvest cells at 4000rpm, 10 minutes, at 0ºC. Discard supernatant.

8. Resuspend cells in 1ml of cold, sterile dH_20 by swirling tubes on ice. Add cold, sterile dH_20 to a final volume of 10ml.

9. Centrifuge 4000rpm, 10 minutes, at 0ºC. Discard supernatant.

10. Repeat steps 8-9 once more for a total of 2 washes.

11. Resuspend the final pellet in 200ul of cold, sterile dH_20.

12. Electroporate cells with the PCR product. We employ a BioRad Gene Pulser Xcell system with a 0.2cm cuvette containing 25µl of cells and 200ηg of PCR product. We electroporate at 2.5kV, 200Ω and 25µF. Under these conditions the time constant is approximately 5.0ms.

13. Add 1ml of L-Broth to the electroporated cells and transfer to a 14ml round bottom tube.

14. Incubate at 32ºC, 225rpm, 1hr.

15. Plate 100 µl and 100 µl of a 1/10 dilution of the cells on L-agar containing 50µg of spectinomycin/ml and incubate 24-48hrs at 32ºC.

16. Prepare plasmids and confirm that the construct is correct.

<u>Construction of a deletion/insertion mutant in NTHi</u>

The plasmid construct is linearized and transformed into NTHi using the MIV transformation method [1]. If counter selection with streptomycin is to be used, a streptomycin resistant NTHi strain with a mutation in the *rpsL* gene is employed. After transformation, mutants are selected on chocolate agar containing 200µg of spectinomycin/ml after growth at 37ºC overnight. If counter selection with streptomycin is used, spectinomycin-resistant clones are screened on chocolate agar containing 1mg of streptomycin/ml. Colonies containing the spectinomycin resistance gene-*rpsL* cassette will be streptomycin-sensitive. The mutation can be confirmed by Southern hybridization and/or PCR.

Construction of an NTHi mutant containing pRSM2814, the FLP recombinase-expressing construct

1. Inoculate NTHi strain (or *rpsL* mutant) into 25ml of sBHI broth (Brain Heart Infusion broth containing NAD and heme at a final concentration of 2μg /ml) in a 250ml flask with cells from plate to an OD at 600ηm of ~0.05 to 0.1.

2. Incubate cells in a 37°C shaking water bath (180rpm) until an OD at 600ηm of 0.3 is reached.

3. Pour cells into 50ml conical and place cells on ice for 30-60 minutes.

4. Pellet cells at 3200 x g, 15 min at 4°C and remove supernatant.

5. Wash pellet 3 x 25ml (1 culture volume of 50% SG) at 3220 x g, 10 min, 4°C. SG is filter sterilized 15%glycerol and 272mM sucrose.

6. Remove final supernatant and resuspend pellet in 250ul of SG to give final cell concentration of 100X the initial concentration.

7. Electroporate cells with the pRSM2814. We employ a BioRad Gene Pulser Xcell system with a 0.1cm cuvette containing 40μl of cells and 500ηg of pRSM2814. We electroporate at 2.5kV, 200Ω and 25μF. Under these conditions the time constant is approximately 5.0ms.

8. Add 1ml of sBHI broth to the cells after electroporation and pipette very gently up and down several times to suspend cells.

9. Place the 1ml cell mixture into a 14ml round bottomed tube and incubate for 1hr at 32°C without shaking.

10. Pipette cells onto a chocolate plate and incubate for 3hrs at 32°C.

11. Wash cells from the chocolate agar plate with 1ml of sBHI and plate dilutions onto chocolate agar containing 20μg of kanamycin/ml.

12. Incubate 24-48hrs at 32°C.

Induction of the FLP Recombinase to remove the spec-*rpsL* cassette followed by curing of pRSM2814

1. Inoculate 10ml of sBHI containing 20μg of kanamycin/ml with the mutant containing pRSM2814 to an OD at 600ηm of 0.05-0.1.

2. Incubate cells at 32ºC, 180rpm, until an OD at 600ηm of ~0.2-0.3 is reached.

3. Add anhydrotetracycline to a final concentration of 200ηg/ml to induce expression of the FLP recombinase.

4. Incubate at 180rpm for 2hrs at 32ºC.

5. Plate 100μl of 10^{-2}, 10^{-4}, 10^{-6} dilutions of cells on chocolate agar containing 1mg/ml of streptomycin or chocolate agar containing no antibiotics if the counter selection is not used.

6. Incubate at 37ºC with 5%CO_2 overnight.

7. Screen clones on chocolate agar containing 200μg spectinomycin/ml and chocolate agar containing 20μg kanamycin/ml to identify clones that have lost the spec-*rpsL* cassette and pRSM2814. Clones with the correct phenotype (spectinomycin- and kanamycin-sensitive) can be maintained on chocolate agar. Mutations should be verified by Southern blot, PCR, and/or sequencing.

Reference:

1. Poje G, Redfield RJ: **Transformation of *Haemophilus influenzae***. *Methods Mol Med* 2003, **71**:57-70.
